# Impact of hypothermia on the biomechanical effect of epithelium-off corneal cross-linking

**DOI:** 10.1186/s40662-021-00229-3

**Published:** 2021-02-09

**Authors:** Hormoz Abdshahzadeh, Reyhaneh Abrishamchi, Emilio A. Torres-Netto, Sabine Kling, Nikki L. Hafezi, Mark Hillen, Farhad Hafezi

**Affiliations:** 1 Ocular Cell Biology Group, Center for Applied Biotechnology and Molecular Medicine, University of Zurich, Winterthurerstrasse 190, 8057 Zurich, Switzerland; 2 ELZA Institute, Dietikon/Zurich, Switzerland; 3 Department of Ophthalmology, Paulista School of Medicine, Federal University of Sao Paulo, Sao Paulo, Brazil; 4 Faculty of Medicine, University of Geneva, Geneva, Switzerland; 5 OPTIC-team, Computer Vision Laboratory, ETH Zurich, Zurich, Switzerland; 6 Department of Ophthalmology, University of Wenzhou, Wenzhou, China; 7 USC Roski Eye Institute, USC Los Angeles, Los Angeles, CA USA

**Keywords:** Corneal cross-linking, CXL, Keratoconus, Temperature, Oxygen diffusion, Hypothermia

## Abstract

**Background:**

The corneal cross-linking (CXL) photochemical reaction is essentially dependent on oxygen and hypothermia, which usually leads to higher dissolved oxygen levels in tissues, with potentially greater oxygen availability for treatment. Here, we evaluate whether a reduction of corneal temperature during CXL may increase oxygen availability and therefore enhance the CXL biomechanical stiffening effect in ex vivo porcine corneas.

**Methods:**

One hundred and twelve porcine corneas had their epithelium manually debrided before being soaked with 0.1% hypo-osmolaric riboflavin. These corneas were equally assigned to one of four groups. Groups 2 and 4 underwent accelerated epithelium-off CXL using 9 mW/cm^2^ irradiance for 10 min, performed either in a cold room temperature (group 2, 4 °C) or at standard room temperature (group 4, 24 °C). Groups 1 and 3 served as non-cross-linked, temperature-matched controls. Using a stress-strain extensometer, the elastic moduli of 5-mm wide corneal strips were analyzed as an indicator of corneal stiffness.

**Results:**

Accelerated epithelium-off CXL led to significant increases in the elastic modulus between 1 and 5% of strain when compared to non-cross-linked controls (*P* < 0.05), both at 4 °C (1.40 ± 0.22 vs 1.23 ± 0.18 N/mm) and 24 °C (1.42 ± 0.15 vs 1.19 ± 0.11 N/mm). However, no significant difference was found between control groups (*P* = 0.846) or between groups in which CXL was performed at low or standard room temperature (*P* = 0.969).

**Conclusions:**

Although initial oxygen availability should be increased under hypothermic conditions, it does not appear to play a significant role in the biomechanical strengthening effect of epithelium-off CXL accelerated protocols in ex vivo porcine corneas.

## Background

Corneal cross-linking (CXL) with riboflavin and ultraviolet (UV)-A light is able to successfully halt the progression of progressive forms of keratoconus [1, 2] and other corneal ectasias [3, 4]. CXL stiffens the cornea through a photochemical process [5, 6], in which oxygen radicals induce the formation of additional covalent bonds between collagen fibrils and proteoglycan core proteins [7].

**Fig. 1:**
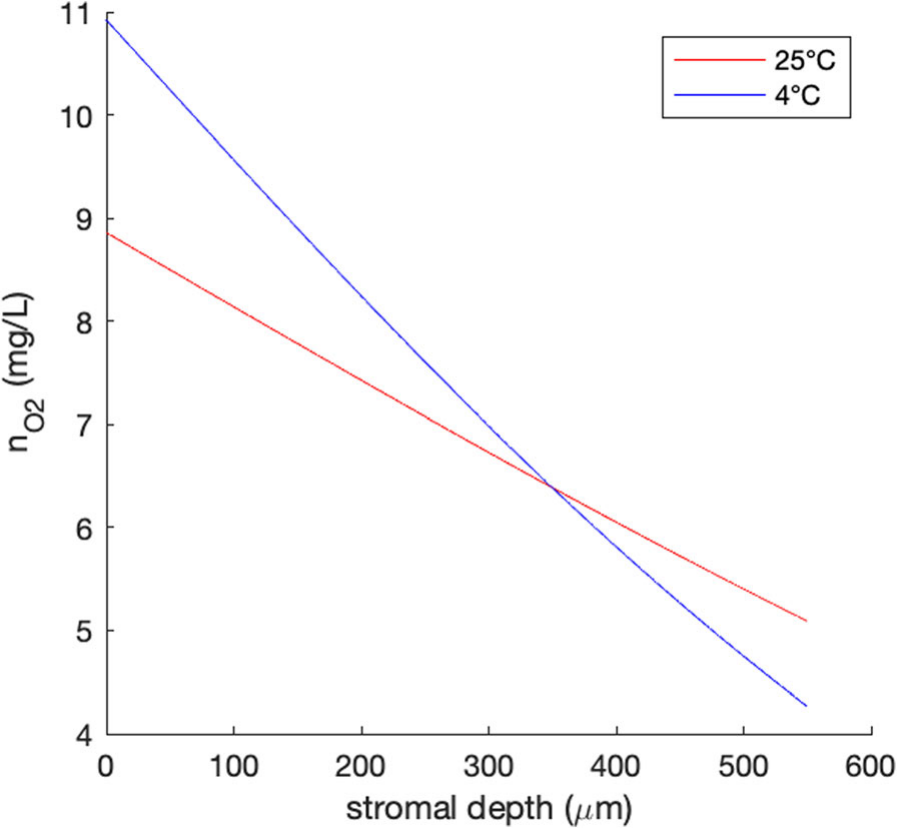
Oxygen concentration after 20 min of diffusion into corneal tissue at 4 and 25 °C

In 2013, our group showed for the first time that the photochemical reaction underlying CXL is essentially dependent on oxygen [8, 9]. This dependence on oxygen may explain numerous recent CXL protocols, either with a lower stiffening effect whenever the availability of oxygen is lower - as in protocols that maintain the epithelium intact (epi-on) and with contact lens-assisted CXL [8, 10], or with a greater stiffening effect whenever the availability of oxygen is higher - as in thin corneas [8]. At the same time, several elements suggest a potential dependency of oxygen availability on temperature: on the one hand, hypothermia leads to higher levels of dissolved oxygen (oxygen tension) in water [11] and the cornea is composed of more than 70% of water [12]. For example, water at 4 °C could hold 10.92 mg/L oxygen, but only 8.68 mg/L at 21 °C. On the other hand, the oxygen diffusion coefficient D_O2_ in tissue is affected by temperature, with slower diffusion rates at lower temperatures [13]. The diffusion coefficient into corneal tissue was reported to be 4 × 10^− 6^ cm^2^/s at 25 °C and 6 × 10^− 6^ cm^2^/s at 35 °C [14]. An exponential relation between *D*_*O2*_ and temperature *T* (*D*_*O*2_ = *C* ∙ *e*^*b* ∙ *T*^), with *b* = 4.4%/°C has been reported in the hamster retractor muscle, where *C* is a constant [13]. Assuming the same exponential relation, *b* can be estimated for cornea from previous experimental data (4.05%/°C). Accordingly, at 4 °C, an oxygen diffusion coefficient of 1.7 × 10^− 6^ cm^2^/s into corneal tissue may be expected. Overall oxygen availability within corneal tissue can be described by Fick’s second law of diffusion; one dimension (i.e., along corneal depth *x*) can be expressed for a given time *t* as: $\left(x,t\right)={n}_0\bullet \mathit{\operatorname{erfc}}\left(\frac{x}{2\sqrt{Dt}}\right)$, where *n*_*0*_ is the oxygen concentration at the surface, and *D* the diffusion coefficient. Applying this equation to estimate the oxygen concentration between 0 and 550 μm stromal depth, we noticed that after 20 min of diffusion, there is on average 7.39 mg/L oxygen available at 4 °C, but only 6.93 mg/L at 25 °C (Fig. 1).

Routine CXL procedures are normally conducted in ambient temperature conditions, at a typical corneal surface temperature of 20 – 25 °C. We hypothesized that reducing the room temperature, and therefore the corneal surface temperature, might be a way to increase oxygen availability and potentially increase biomechanical stiffening effect of CXL. Such a reduction of surface stromal temperature could be clinically achieved by rinsing the corneal surface with chilled balanced salt solution (BSS), for example. We therefore investigated here whether hypothermia would modulate the biomechanical effect of the cross-linking process.

## Methods

### Specimens

Freshly enucleated porcine eyes with intact epithelium (*n* = 112) from young adult pigs, aged between 6 to 8 months old (that had first been sacrificed for food production) were obtained from the local abattoir and used within 6 h. Eyes were allocated equally to four groups, *n* = 28 per group. Eyes from groups 1 and 2 were always handled in a refrigerated, cold temperature-controlled room at 4 °C. Eyes from groups 3 and 4 were always handled in a standard temperature-controlled room at 24 °C.

### Experimental protocols

All corneas of all groups had their epithelium removed manually with a surgical blade, and then, were soaked with 0.1% iso-osmolar riboflavin (Streuli Pharma, Uznach, Switzerland) for 20 min. Corneas from groups 1 and 3 served as non-CXL controls. In the corneas of groups 2 and 4, an accelerated epithelium-off protocol CXL was performed as follows. After soaking, corneas in groups 2 and 4 were exposed to UV-A irradiation (365 nm; CCL-Vario Crosslinking; Peschke MediTrade GmbH, Zurich, Switzerland) at an intensity of 9 mW/cm^2^ for 10 min (Table 1). In all groups, throughout soaking and irradiation, corneal surface temperature was continuously monitored using an infrared laser thermometer (Extech Instruments, FLIR Commercial Systems Inc., Nashua, USA).

**Table 1:** Corneal cross-linking experimental protocol specifications used in both groups

Parameter	Experimental groups
Treatment target	Experiment
Fluence (total; J/cm^2^)	5.4
Soak time (minutes) and interval	20 (q2)
Intensity (mW/cm^2^)	9
Treatment time (minutes)	10
Epithelium status	Off
Chromophore	0.1% riboflavin
Light source	CCL-Vario
Irradiation mode	Continuous

### Sample preparations

After removing the corneoscleral button from the globe, two corneoscleral strips (5 mm width, full thickness) were prepared centrally in the horizontal axis of each eye. Four millimeters at the end of each strip were dedicated to fixation, leaving approximately 11 mm of central corneal strip length. Since hydration status and corneal temperature could impact biomechanical measures, such factors were controlled. Prior to stress-strain measurements, all samples were kept in a 400 mOsml/l balanced saline solution in room temperature for 10 min.

### Biomechanical measurements

The tensile strength of all corneas was analyzed using a stress-strain extensometer (Z0.5; Zwick GmbH & Co., Ulm, Germany). The Z0.5 is a classical extensometer that measures the real-time force in Newtons exerted by the arm that holds the specimen; the speed at which the force is applied can be controlled by the user. The conversion from force to stress was calculated from the thickness and width of the specimen. The arm speed was 2 mm per minute in the conditioning cycles and the stress-strain testing was performed over a 4 mm length. The biomechanical analysis included testing up to a force of 4 N. The slope of the stress-strain curve corresponds to the tangent elastic modulus and was determined between 1 and 5% of strain. This range was selected as it showed a linear relation in the stress-strain diagram.

### Statistical analysis

Statistical analysis was performed using GraphPad Prism 8.0 software package (GraphPad Software, Zurich, Switzerland) and Microsoft Excel (Excel 11 for Mac; Microsoft Corporation, Redmond, WA). Normal distribution was confirmed with the Shapiro–Wilk, Kolmogorov-Smirnov and D’Agostino & Pearson tests. Due normality in all groups, one-way ANOVA test with statistical significance was used to determine significant differences between groups, with a confidence interval of 95%. Statistical significance was considered whenever the *p* value was 0.05.

## Results

Corneal surface temperatures varied between 23 and 25 °C for the standard temperature-controlled room groups and between 3 and 5 °C for the cold temperature-controlled room groups.

**Fig. 2:**
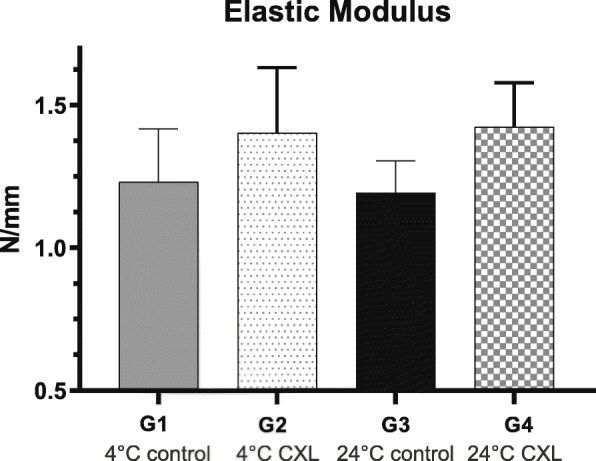
Representation of the tangential elastic modulus in all groups studied: low room temperature riboflavin-soaked corneas without irradiation (Group 1, 4 °C control), low room temperature riboflavin-soaked corneas irradiated with 9 mW/cm^2^ for 10 min (Group 2, 4 °C CXL), standard room temperature riboflavin-soaked corneas without irradiation (Group 3, 24 °C control) and standard room temperature riboflavin-soaked corneas irradiated with 9 mW/cm^2^ for 10 min (Group 4, 24 °C CXL)

Figure 2 shows the Elastic Modulus in all groups. Accelerated epithelium-off CXL led to significant increases in the elastic modulus between 1 and 5% of strain when compared to non-cross-linked controls, both at 4 °C (1.40 ± 0.22 vs 1.23 ± 0.18 N/mm, *P* = 0.006) and 24 °C (1.42 ± 0.15 vs 1.19 ± 0.11 N/mm, *P* < 0.001). No significant difference was found in the Elastic Modulus between control groups 1 and 3 (*P* = 0.846) or between groups 2 and 4, in which CXL was performed (*P* = 0.969) at the low and standard room temperature, respectively.

## Discussion

Considering that (a) the availability of tissue oxygen may vary at different temperatures, and (b) oxygen is essential for CXL reactions, this study aimed to assess whether a reduction in the surface temperature of the cornea during CXL could cause an additional stiffening effect. However, a temperature-dependent effect on CXL could not be measured: no significant differences were found between corneas treated with CXL at cold (4 °C) or at standard (24 °C) temperature-controlled rooms.

**Fig. 3:**
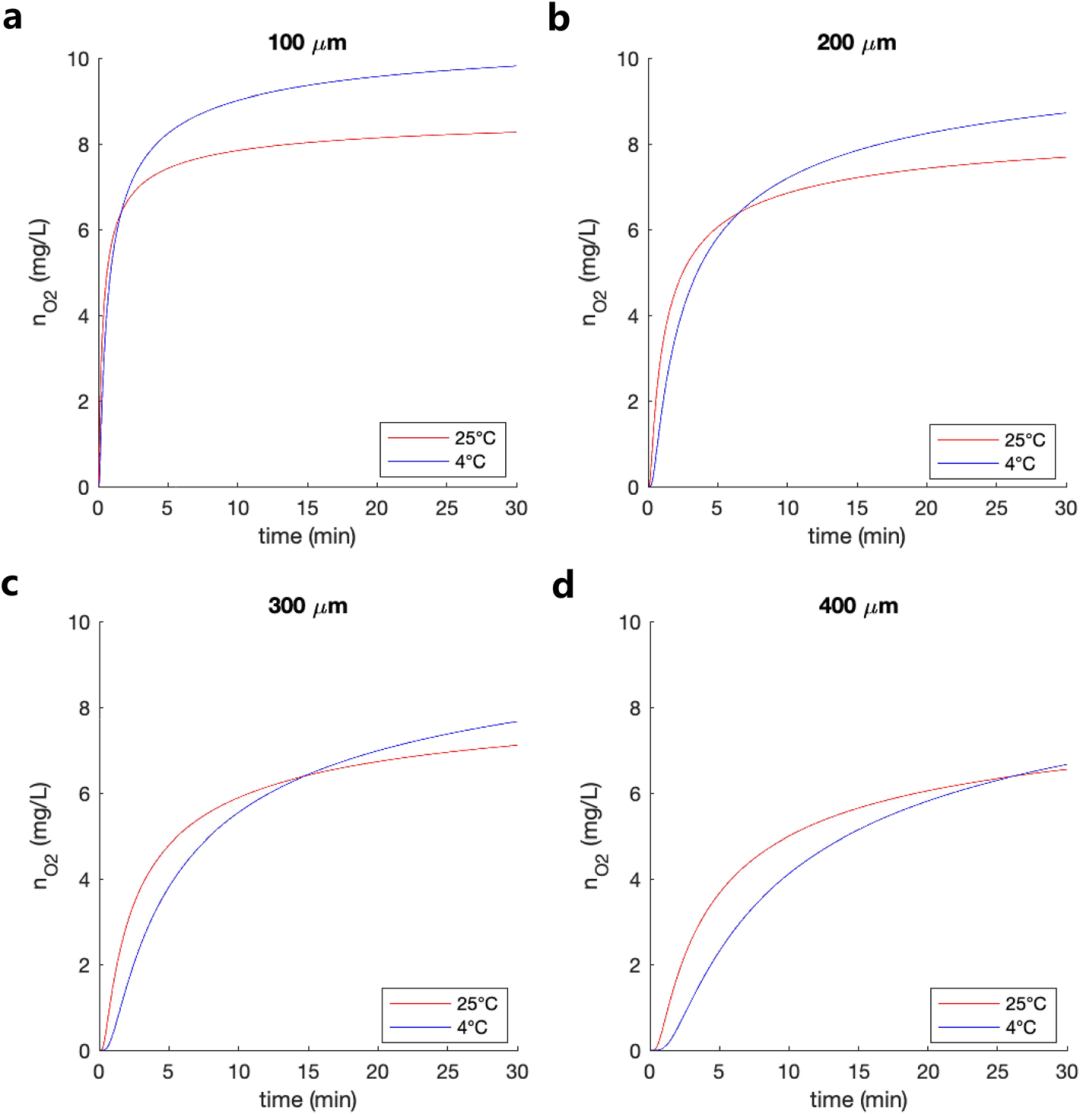
Oxygen concentration at different stromal depths after 20 min of diffusion

When interpreting this result in more detail, we need to consider that CXL is based on a photochemical process that involves a chromophore (riboflavin, vitamin B_2_) activated by an energy source (UV-A light) [5]. According to Fick’s law, higher oxygen concentrations and faster replenishment can be expected in the most anterior stroma. Apart from a higher UV absorption in the anterior stroma, this is possibly an additional reason why CXL is only effective up to a stromal depth of approximately 350 μm. When accounting for the effect of temperature on oxygen diffusion, we found that the imbalance of oxygen availability between anterior and posterior stromal tissue is increased at lower temperatures (Fig. 3). In this study, no significant differences were observed between 4 and 24 °C cross-linking conditions, suggesting that the initial oxygen concentration in the anterior stroma is less relevant for the biomechanical stiffening effect.

In 2013, our group demonstrated that oxygen availability in the corneal stroma is an essential and limiting factor for the effectiveness of biomechanical stiffening in CXL [9]. Later, it has been shown that as the photochemical reaction proceeds, oxygen is consumed, and tissue oxygenation decreases within a few seconds after the onset of UV-A light, but only gets replenished minutes after UV-A irradiation has been stopped [14]. When looking at the first seconds of oxygen diffusion into the cornea, higher temperatures are favorable according to Fick’s law, because of the higher diffusion coefficient. In the context of our results, this likely indicates that throughout the irradiation period, more oxygen might be diffusing into the cornea at higher temperatures. The benefit of a higher oxygen concentration at the beginning of the irradiation at lower temperatures is likely comparable to the benefit of a higher oxygen flux into the tissue during irradiation at higher temperatures.

The fact that there was a tendency of greater stiffening effect when CXL was performed at standard temperature rather than at cold temperature – the normalized CXL stiffening effect was 19% at 24 °C and 14% at 24 °C – might indicate that the continuous oxygen flux during irradiation is potentially more important than initial oxygen saturation in terms of biomechanical stiffening. Further investigations in this direction are needed to verify this hypothesis. If it holds true, conventional CXL with 30 min irradiation time should benefit more from higher temperatures than accelerated CXL, which was used in this study.

This study has potential limitations. Whereas the scope of this study was to investigate the effect of temperature on oxygen diffusion, changing ambient temperature might not only affect the rate of oxygen diffusion, but also the reaction rates of enzymes like the lysyl oxidase (LOX)-mediated pathway [15]. The role of such enzymes in ectatic diseases, however, is not fully understood and there is still a lack of evidence that this could directly lead to KC development [15]. Additionally, the temperature condition used in this study would not entirely represent clinical conditions. Although the objective here was to verify the basic principles rather than immediately develop a clinical application, corneal cooling could potentially be achieved by applying cold BSS.

## Conclusions

In conclusion, while oxygen plays an essential role in corneal cross-linking, our results suggest that corneal stromal temperature does not affect the biomechanical effect of epithelium-off accelerated CXL in ex vivo porcine corneas.

## Data Availability

The data will be made available on request.

## References

[1] Wollensak G, Spörl E, Seiler T (2003). Treatment of keratoconus by collagen cross linking. Ophthalmologe..

[2] Raiskup F, Theuring A, Pillunat LE, Spoerl E (2015). Corneal collagen crosslinking with riboflavin and ultraviolet-A light in progressive keratoconus: ten-year results. J Cataract Refract Surg.

[3] Hafezi F, Kanellopoulos J, Wiltfang R, Seiler T (2007). Corneal collagen crosslinking with riboflavin and ultraviolet A to treat induced keratectasia after laser in situ keratomileusis. J Cataract Refract Surg.

[4] Richoz O, Mavrakanas N, Pajic B, Hafezi F (2013). Corneal collagen cross-linking for ectasia after LASIK and photorefractive keratectomy: long-term results. Ophthalmology..

[5] Spörl E, Huhle M, Kasper M, Seiler T (1997). Increased rigidity of the cornea caused by intrastromal cross-linking. Ophthalmologe..

[6] Choi S, Shin JH, Cheong Y, Jin KH, Park HK (2013). Structural and biomechanical effects of photooxidative collagen cross-linking with photosensitizer riboflavin and 370 nm UVA light on human corneoscleral tissues. Microsc Microanal.

[7] Hayes S, Kamma-Lorger CS, Boote C, Young RD, Quantock AJ, Rost A (2013). The effect of riboflavin/UVA collagen cross-linking therapy on the structure and hydrodynamic behaviour of the ungulate and rabbit corneal stroma. PLoS One.

[8] Kling S, Richoz O, Hammer A, Tabibian D, Jacob S, Agarwal A (2015). Increased biomechanical efficacy of corneal cross-linking in thin corneas due to higher oxygen availability. J Refract Surg.

[9] Richoz O, Hammer A, Tabibian D, Gatzioufas Z, Hafezi F (2013). The biomechanical effect of corneal collagen cross-linking (CXL) with riboflavin and UV-A is oxygen dependent. Transl Vis Sci Technol.

[10] Torres-Netto EA, Kling S, Hafezi N, Vinciguerra P, Randleman JB, Hafezi F (2018). Oxygen diffusion may limit the biomechanical effectiveness of iontophoresis-assisted transepithelial corneal cross-linking. J Refract Surg.

[11] Wetzel RG (2001). Limnology: lake and river ecosystems.

[12] Taylor ZD, Garritano J, Sung S, Bajwa N, Bennett DB, Nowroozi B (2015). THz and mm-wave sensing of corneal tissue water content: electromagnetic modeling and analysis. IEEE Trans Terahertz Sci Technol.

[13] Bentley TB, Pittman RN (1997). Influence of temperature on oxygen diffusion in hamster retractor muscle. Am J Phys.

[14] Kamaev P, Friedman MD, Sherr E, Muller D (2012). Photochemical kinetics of corneal cross-linking with riboflavin. Invest Ophthalmol Vis Sci.

[15] Siemens J, Kamm GB (2018). Cellular populations and thermosensing mechanisms of the hypothalamic thermoregulatory center. Pflugers Arch.

